# Schizoaffective disorder about 57 cases

**DOI:** 10.1192/j.eurpsy.2021.2147

**Published:** 2021-08-13

**Authors:** I. Kammoun

**Affiliations:** Psychiatry G, RAZI UHC, Tunis, Tunisia

**Keywords:** psychiatric interview, schizoaffective disorder, personality disorder

## Abstract

**Introduction:**

Schizoaffective disorder remains relatively unknown today compared to other psychiatric disorders. This disorder is however recognized by the international medical classifications DSM 5 and mainly affects many people.

**Objectives:**

Describe the socio-demographic and contextual clinical characteristics of patients with schizoaffective disorder

**Methods:**

We conducted a descriptive retrospective study including patients with schizoaffective disorder (DSM 5) in the psychiatric department G at Razi hospital and who were hospitalized for a period of 1 year from 1 January to 21 December 2020. We collected 57 patients.

**Results:**

The average age of our sample is 40.16 years. The majority of patients (75.4%) were single and the school level did not exceed secondary studies in 64.9% of cases. Most of these patients were unemployed previously working as a day laborer in 47.4%. In addition, the type of schizoaffective disorder was dominated by the bipolar type (94.7%). These patients had a personality disorder in 26.3% mainly schizoid. The psychiatric interview of these patients revealed irritable mood in 47.4%, inappropriate affects in 59.6%, speech of a maniac in 52.6%, delusions of persecution and grandeur in 70.2% with intuitive mechanism (47.4%) and hallucinatory (auditory 45.6%). Disorganized behavior in 50% and catatonic behavior in 5.3% Mental automatism and morbid rationalism in 29.8% Insomnia: 94.7% and concentration disorder: 56.1% Type of treatment was the combination of atypical antipsychotics, mood stabilizers and benzodiazepines 33.3% with regular follow-up in 49.1%

**Conclusions:**

Schizoaffective disorder is one of the most misdiagnosed psychiatric disorders in clinical practice and the need to know its characteristics is a necessity.

**Disclosure:**

No significant relationships.

